# Advancing Seabird Diet Studies Through Buccal Swabbing for DNA Metabarcoding

**DOI:** 10.1002/ece3.71606

**Published:** 2025-07-09

**Authors:** Lauren G. Evans, Stephanie M. Harris, Paul M. Thompson, Amy Ellison, Line S. Cordes

**Affiliations:** ^1^ School of Environmental and Natural Sciences Bangor University Bangor UK; ^2^ School of Ocean Sciences Bangor University Menai Bridge UK; ^3^ School of Environmental Sciences University of Liverpool Liverpool UK; ^4^ Lighthouse Field Station, Institute of Biological and Environmental Sciences University of Aberdeen Aberdeen UK; ^5^ Norwegian Institute for Nature Research (NINA) Trondheim Norway

**Keywords:** buccal swabbing, diet studies, DNA metabarcoding, prey, Procellariiform, seabird

## Abstract

Seabirds are often considered sentinels of ocean health, making dietary studies crucial not only for understanding their ecology and marine trophic webs, but also for informing conservation efforts and detecting ecosystem changes that may threaten biodiversity. However, determining the diet of wide‐ranging, pelagic species is challenging, and there is a series of limitations associated with current techniques. In this study, we investigated buccal swabbing and DNA metabarcoding as a combined method to determine the diet of Manx shearwaters ( *Puffinus puffinus* ) and northern fulmars ( *Fulmarus glacialis* ) during different stages of the breeding season. We detected 14 fish taxa, with prey DNA successfully amplified in 68% of Manx shearwater samples and 28% of northern fulmar samples. We suggest that differences in amplification success between sample types are due to the time elapsed between feeding and swabbing when sampling various breeding stages. We present the first species‐level dietary data for chick‐provisioning Manx shearwaters and reveal a potential reliance on calorie‐dense European sprat ( *Sprattus sprattus* ). In addition to identifying two fish taxa not previously documented as northern fulmar prey, our results highlight the continued importance of fishery discard species in their diet during the breeding season. This study suggests both species may be sensitive to shifts in prey availability and fishing practices and demonstrates the utility of buccal swabbing for DNA metabarcoding as a minimally invasive tool for dietary analysis in pelagic seabirds.

## Graphical abstract

Determining the diet of wide‐ranging, pelagic seabirds is challenging. In this study, we investigated buccal swabbing and DNA metabarcoding to describe the diet of Manx shearwaters ( *Puffinus puffinus* ) and northern fulmars ( *Fulmarus glacialis* ). We reveal a potential dietary specialism in shearwaters and highlight the continued importance of fishery discard species in the diet of fulmars.

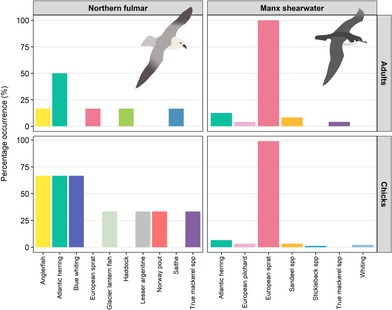

## 1 Introduction

Understanding diet is fundamental to ecology and conservation, shaping species' foraging strategies, survival and reproductive success (Lourenço et al. 2015; Sky et al. 2024). Dietary studies can provide insights into the health and viability of animal populations and serve as a crucial tool for monitoring changes in lower trophic levels and broader ecosystem shifts (Birnie‐Gauvin et al. 2017). Investigating what animals eat and how their diets respond to environmental pressures enhances our understanding of species interactions, ecosystem stability and biodiversity conservation (Nielsen et al. 2017). For species of conservation concern, assessing diet can help identify key prey resources and potential threats (Wolf and Ripple 2016), while for wide‐ranging or indicator species, dietary data can offer valuable information on prey availability and food web structure (Ford et al. 2016; Romero et al. 2021). However, accurately determining the diet of elusive or wide‐ranging species, such as marine top predators, remains particularly challenging (Barrett et al. 2007).

Seabirds are among the most threatened groups of birds globally (Dias et al. 2019), facing pressures from climate change, extreme weather events, invasive species, overfishing and plastic pollution (Cox et al. 2018). Although many studies focus on seabird demography, fewer identify the underlying mechanistic drivers of population change (Scales et al. 2014; Bost et al. 2015). Understanding how variation in seabird diet and foraging behaviour influences reproductive success is crucial for predicting the impacts of shifts in lower marine trophic levels on seabird populations (Lorentsen et al. 2019; Fayet et al. 2021). Additionally, as apex predators, dietary studies of seabirds serve as important indicators of changes in prey availability and broader ecosystem dynamics, making them valuable tools for monitoring regime shifts (Waap 2015; Alonso et al. 2018; Hazen et al. 2019). Seabirds are therefore widely considered sentinels of ocean health (Brisson‐Curadeau et al. 2017; McInnes, Alderman, et al. 2017; Alonso et al. 2018; Clucas et al. 2024). Consequently, seabird diet studies not only enhance our understanding of species ecology but also contribute to broader research on marine food webs, ecosystem dynamics, and top‐down versus bottom‐up regulatory processes (Cherel and Weimerskirch 1995; Menning et al. 2023).

Determining seabird diet is often challenging and the efficacy of current techniques are hampered by a series of limitations. Traditionally, diet studies relied on visual identification of prey species from pellets, regurgitates or stomach contents, often obtained by collecting voluntary regurgitates, stomach flushing or lethal sampling (Brown et al. 1981; Phillips et al. 1999; Polito et al. 2011). Stomach content analysis is invasive (Barrett et al. 2007), and subject to bias due to differential digestion and retention times of tissues (Deagle et al. 2007). Morphological analysis of remnant hard‐parts or semi‐digested tissues to species‐level relies on taxonomic expertise but even then, is sometimes not possible (Barrett et al. 2007; Alonso et al. 2014). Stable isotope analysis can evaluate trophic position, region of feeding and the relative contribution of nearshore or offshore prey in the diet using the ratio of nitrogen‐15 (^15^N) to nitrogen‐14 (^14^N) and carbon‐13 (^13^C) to carbon‐12 (^12^C) in tissue samples (Barrett et al. 2007; Karnovsky et al. 2012). Similarly, analysis of fatty acids stored in blood, adipose tissue or Procellariiform stomach oil can identify trophic biomarkers or differences in diet between groups (Karnovsky et al. 2012; Owen et al. 2013). Both these techniques, however, rely on an understanding of a species' foraging range, potential prey and their distinct isotopic or fatty acid signatures (Polito et al. 2011; Karnovsky et al. 2012). The timescale of dietary information provided by both methods varies by tissue type, ranging from a few days for rapidly metabolised tissues like blood plasma, to several months for feathers or adipose tissue (Barrett et al. 2007). As a result, both stable isotope and fatty acid analysis can only provide coarse dietary information and are limited in their ability to investigate diet at the finer scale (Deagle et al. 2010; Jarman et al. 2013).

DNA metabarcoding offers a cost‐effective and minimally invasive method for accurately determining diet (Symondson 2002; Barrett et al. 2007) and its application to faecal samples in marine organisms has been revolutionary (Menning et al. 2023). However, the use of faecal samples still faces a series of limitations: low amplification success due to the presence of PCR inhibitory substances like urea, differential digestion and retention times of organisms or tissues, and environmental contamination (Deagle et al. 2010; Karnovsky et al. 2012; Jarman et al. 2013; Oehm et al. 2017; Nota et al. 2019; Siddiqi‐Davies et al. 2025). Collection of faecal samples requires either opportunistically gathering available faeces from a colony (where a sample cannot be traced to an individual), waiting for an individual to defecate (a time‐consuming process) or cloacal swabbing (which requires extensive handling and can be stressful for an individual) (McInnes, Alderman, et al. 2017; Fayet et al. 2021; Lefort et al. 2022). A single faecal sample also likely only represents a small proportion of a meal (Oehm et al. 2017).

Buccal swabbing poses a novel alternative to faecal sampling to investigate diet in seabirds. Although primarily used to sample DNA for microsatellite marker description or molecular sexing (Yannic et al. 2011; Pande et al. 2018; Vilstrup et al. 2018), DNA metabarcoding of buccal swabs has been successfully used to determine the diet of hen harriers (*Cirus cyaneus*) (Nota et al. 2019), rough‐legged hawks ( *Buteo lagopus* ) (Paprocki et al. 2024) and green sea turtles ( *Chelonia mydas* ) (Díaz‐Abad et al. 2022). In comparison to faecal samples, DNA obtained from buccal swabs should be less degraded, extracted alongside fewer PCR inhibitors, and less affected by organism or tissue‐specific biases during digestion (Barrett et al. 2007; Oehm et al. 2017; Nota et al. 2019). Although small amounts of DNA from previous meals will likely persist in the mouth, buccal swabbing should primarily capture prey DNA from the most recent meal. Buccal swabbing also enables the targeted sampling of specific, individual animals. The ability to attribute diet samples to known individual birds facilitates answering a broad array of important outstanding questions in ecology, such as how diet varies with age, sex and other characteristics, or in combination with biologging, how diet varies with foraging locations and behavior.

For many breeding seabirds, diet can be determined by observing whole prey carried in adults' bills and provided to chicks (Clucas et al. 2024). As an adaptation to long, wide‐ranging foraging trips and to reduce the time and energetic costs of transporting whole prey back to the colony, some groups of seabirds, such as Procellariiformes, have evolved to feed their chicks semi‐digested food (Brooke 1990; Warham 1997). This strategy involves delivering food directly into the mouth of the chick via regurgitation, making visual identification of semi‐regurgitated prey difficult. Given these challenges, buccal swabbing and DNA metabarcoding offer a particularly promising technique with which to explore the foraging ecology and diet of Procellariiform seabirds.

This study aims to assess whether buccal swabbing and DNA metabarcoding can be used for dietary analysis of two species of Procellariiformes: Manx shearwaters ( *Puffinus puffinus* ) and northern fulmars ( *Fulmarus glacialis* ). We investigate the use of these techniques across different breeding stages: incubation, when individuals undertake long foraging trips without regurgitation (northern fulmars), chick provisioning, when adults have recently regurgitated food for their chicks (Manx shearwaters) and in chicks (both species). Additionally, for Manx shearwaters, we collected diet samples from adults and their chicks on the same night to assess whether chick diet accurately reflects the meals provided by chick‐provisioning adults.

## 2 Methods

### 2.1 Study Species

Manx shearwaters are a medium‐sized shearwater. The United Kingdom hosts around 80% of the world's breeding population (Joint Nature Conservation Committee (JNCC) 2023) and is an important stronghold for this Amber‐listed species of conservation concern (Stanbury et al. 2021). Despite extensive research on their foraging behaviour (Guilford et al. 2008; Fayet et al. 2015; Shoji et al. 2016), there is a significant knowledge gap regarding the diet of Manx shearwaters. It is generally understood that this highly pelagic species feeds predominantly on fish, such as small clupeids and sandeels, and squid, like other shearwaters in the genus *Puffinus* (Brown et al. 1981; Dean et al. 2015; Komura et al. 2018; Siddiqi‐Davies et al. 2025). Traditionally, studies have relied on stomach content analyses obtained via stomach flushing or lethal sampling, with most data from the non‐breeding season (Furness 1994; Petry et al. 2008; Thompson 1987). More recently, DNA metabarcoding has been used to analyse faecal samples from Manx shearwaters during the pre‐laying and incubation periods. This study identified six different fish species but had a low success rate, detecting prey DNA in 12% of samples, likely due to degradation of prey DNA in faecal samples (Siddiqi‐Davies et al. 2025).

Northern fulmars (hereafter “fulmar”) are fulmarine petrels which breed widely across the northern hemisphere, although the British‐breeding population is Amber‐listed (Stanbury et al. 2021). Fulmars are predominantly generalist surface feeders (Hobson and Welch 1992; Garthe and Furness 2001) with a highly varied diet including, but not limited to, fish, cephalopods, polychaetes, jellyfish, shrimp and plankton (Phillips et al. 1999; Mallory et al. 2010). Unlike Manx shearwaters, fulmars are known to associate with fishing vessels, and fishery discards and offal are thought to form a major component of their diet (Darby et al. 2021), especially during the non‐breeding season (Ojowski et al. 2001) and at the southern extremes of their range (Phillips et al. 1999).

### 2.2 Buccal Sampling

**FIGURE 1:**
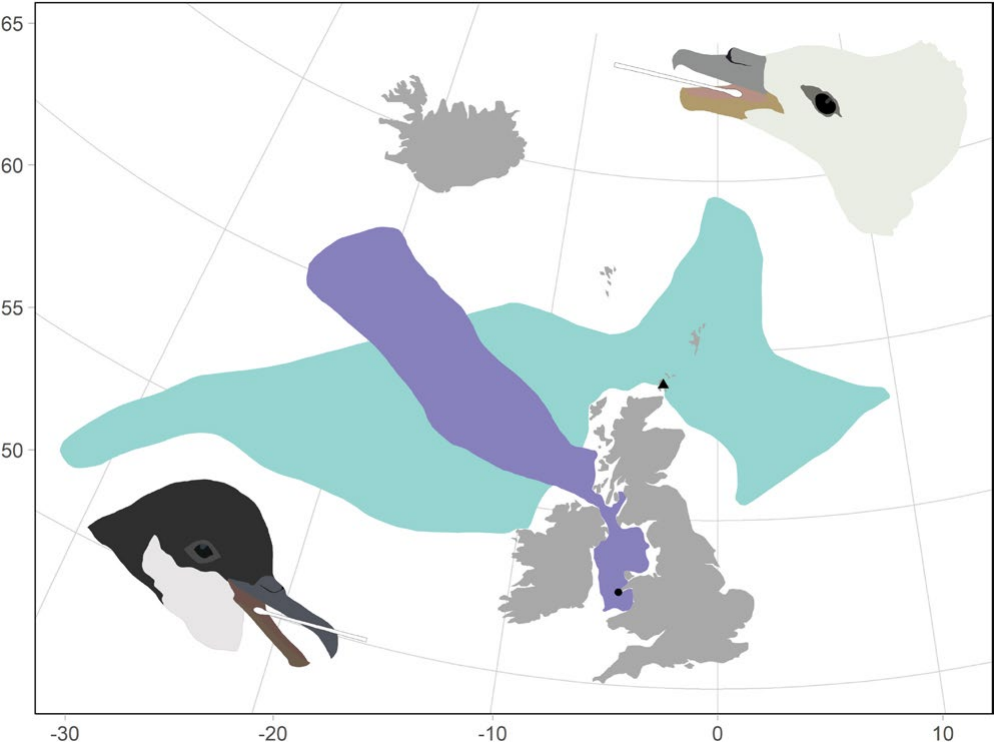
Map showing approximate foraging range of northern fulmars (green) and Manx shearwaters (purple). Fulmar foraging ranges were derived from GPS tracking data collected during incubation and chick‐rearing periods from 2009 to 2017 from individuals tagged on Eynhallow, Orkney (black triangle) (Edwards et al. 2016; Darby et al. 2021). Manx shearwater foraging ranges were derived from GPS tracking data collected on Bardsey Island (black circle) during chick‐rearing period of 2024 (unpublished data).

Buccal swabbing of Manx shearwaters was carried out between early July and early August 2024 on Bardsey Island, Wales (52.759° N, 4.786° W) (Figure 1). After returning to the colony at night and being given sufficient time to feed their chicks, adult birds were gently removed from their burrows. Buccal swabs were collected from 36 adult birds (one per individual) and 133 samples were obtained from 36 chicks, with a mean of 3.61 samples per chick (Range: 1–9) throughout the chick‐rearing period.

Buccal swabbing of adult fulmars took place in early June 2024 on Eynhallow, Orkney, Scotland (59.143° N, 3.120° W) (Figure 1). Fifteen adult fulmars were caught on the nest whilst incubating, and a further seven individuals of unknown breeding status were caught in the air using a fleyg net. In August 2024, ten chicks from nests at the same colony were swabbed, with one swab sample collected per chick, as part of ongoing ringing and nest monitoring at this colony (see Dunnet 1991).

For both species, samples were collected using Medical Wire Dryswab sterile polyester tip swabs, with a 3 mm width bud and flexible plastic shaft. The buccal cavity of the adults and chicks were swabbed by gently rotating the swab behind the tongue for 10 s. Swab tips were then cut off into Eppendorf tubes and frozen at −18°C until DNA extraction. Nitrile gloves were changed between birds to minimise field and cross‐contamination. All work was carried out with approval from the Bangor University's Animal Welfare and Ethical Review Body (AWERB) and was approved by the British Trust for Ornithology Special Methods Permit Technical Panel (permit numbers: 12,267 and 12,481). The entire handling process took less than 10 min. For Manx shearwaters, chick survival to the end of the study period was greater in manipulated (36 chicks survived from 36 burrows) compared to unmanipulated, control burrows (7 chicks survived from 8 burrows) suggesting no detrimental effects of buccal swabbing.

### 2.3 DNA Metabarcoding

All metabarcoding steps were conducted in laboratory spaces with physical separation between pre‐ and post‐PCR areas. All work surfaces and equipment were sterilised using 10% bleach followed by ultraviolet (UV) irradiation. DNA was extracted from buccal swabs using QIAGEN DNeasy Blood and Tissue Extraction Kit following the manufacturers' instructions with minor modification; elution in 100 μL Buffer AE and re‐eluting to increase overall DNA yield. Cut‐off swab tips were incubated with lysis Buffer ATL and Proteinase K and removed at the end of the incubation step with sterile tweezers. Extraction blanks containing no tissue were included alongside all DNA extractions. Three different primer sets targeting fish, cephalopod and decapod DNA were multiplexed in a 1‐step PCR approach (Table S1) (Kleinschmidt et al. 2019; Komai et al. 2019; De Jonge et al. 2021). These primers were chosen to target putative prey items within the diet samples (Thompson 1987; Garthe et al. 2004). Both 16S and 18S rRNA markers are commonly used to target fragmented DNA within eDNA metabarcoding studies as hypervariable regions offer high taxonomic resolution whilst flanking conserved regions are suitable for primer design (Waap 2015; De Jonge et al. 2021; Querejeta et al. 2023). Efficacy of primer multiplexing was tested in a pilot study by running PCRs with each primer set both individually and in combination, using swab samples and a diluted mock community of non‐UK species DNA derived from pure tissue samples (Basa *Pangasius bocourti* , Humboldt squid *Dosidicus gigas* and whiteleg shrimp *Penaeus vannamei* ) mixed at equimolar concentrations and run alongside swab samples. Forty‐eight 12 bp multiplex identifier (MID) tags were incorporated into forward and reverse primers to assign DNA sequences to their respective samples (for full details of MID tag sequences see project accession number PJRE88873). We used unique dual indexes for each sample so that any erroneously tagged reads (e.g., via PCR chimeras or tag‐jumping during library preparation (Rodriguez‐Martinez et al. 2022)) would be removed during subsequent de‐multiplexing.

PCRs were carried out in 25 μL reactions composed of 12.5 μL QIAGEN Multiplex PCR Master Mix, 1 μL of each primer (10 nM), 2 μL of DNA extract, and 8.5 μL ultra‐pure water. PCR positive controls (non‐UK species, as described above), extraction blank controls, and PCR negative controls were run alongside swab samples. PCR cycling was as follows: 95°C for 15 min, followed by 35 cycles of denaturing at 94°C for 30 s, annealing at 58°C for 90 s, extension at 72°C for 45 s, and a final elongation step at 72°C for 5 min (Kleinschmidt et al. 2019).

Amplification success was checked by visually inspecting PCR products on 1.5% agarose gel. If either the positive control failed to amplify, or the PCR negative control amplified, PCRs were repeated. PCR products were quantified using Qubit Broad Range DNA assays (Thermofisher) and pooled in equimolar concentrations. PCR pools were purified using Agencourt AMPure beads. Purified PCR pools were prepared using NEBNext Ultra IIDNA Library Prep Kit and sequenced via Illumina NovaSeq (2 × 250 bp) by Novogene (UK) Company Ltd.

### 2.4 Bioinformatics

We demultiplexed raw sequence reads according to MID tags and trimmed index and primer sequences using *cutadapt* (Martin 2011). We used the DADA2 pipeline (Callahan et al. 2016) to inspect read quality, filter and trim reads (default recommended parameters), denoise (using the makeBinnedQualErr function to account for Novaseq data), merge paired‐end reads, and remove chimeras (Table S2). We then manually inspected putative chimeras for erroneously identified sequences. Amplified sequence variants (ASVs) whose length fell outside the expected amplicon length (FISH2_16S 240‐290 bp; Ceph18S 150‐220 bp; MiDeca 120‐240 bp) were discarded.

ASVs were searched against the National Centre for Biotechnology Information (NCBI) GenBank nucleotide (nt) database using blast (National Center for Biotechnology Information (NCBI) 2024). The top 20 hits (*e*‐value ≤ 0.001, > 70% alignment length) were evaluated by the Assign‐Taxonomy‐with‐BLAST python script using default settings (https://github.com/Joseph7e/Assign‐Taxonomy‐with‐BLAST) to provide final taxonomic assignments.

ASVs with a blast assignment ≥ 97% were identified to species level, ≥ 90% to genus level. We manually performed further filtering to provide robust taxonomic assignment, filtering out unidentified reads and reads originating from bacteria, archaea, plants, mammals, and the birds themselves, so only ASVs considered prey items were retained for further analysis. Lastly, we manually ensured all prey items identified occurred within the known foraging ranges of fulmars or Manx shearwaters, respectively.

Rarefaction curves were plotted using the *vegan* package (Oksanen et al. 2024). For both Manx shearwater and fulmar samples, 500 reads per sample were necessary to capture fish diversity, and we discarded any samples that contained fewer than 500 reads in further analysis. Extraction blanks and PCR negatives were also dropped as they contained no prey DNA sequences, and so field and lab contamination appeared to be negligible.

### 2.5 Diet Metrics

We used two different metrics to describe dietary composition. Percentage occurrence (PO) represents the number of samples containing a given prey item divided by the total number of samples. While PO provides a broad overview of dietary diversity, it can overestimate the importance of rare food items in the diet (Deagle et al. 2019). Relative read abundance (RRA) is the number of reads of a prey item divided by the total number of reads of all prey items in that sample. Average relative read abundance is averaged across all samples for each bird species and is unaffected by variations in sequencing depths among samples (Deagle et al. 2010; Jarman et al. 2013). RRA has long been regarded as a semi‐quantitative metric as the number of reads recovered varies between prey items due to prey tissue DNA density and amplification success (Deagle et al. 2010; Jarman et al. 2013). However, more recent studies have suggested that RRA does show a strong positive correlation with prey biomass consumed and can provide a useful proxy (Clucas et al. 2024; Kennerley et al. 2024).

### 2.6 Statistical Analyses

All analyses were carried out in R Studio version 4.4.2 (R Core Team, 2024). Species accumulation curves with extrapolation were plotted using the *iNEXT* R package and 100 random permutations (Chao et al. 2014; Hsieh et al. 2024).

## 3 Results

Illumina sequencing resulted in 65 million raw reads across all buccal swab samples, and 43.7% of reads were retained after filtering, denoising, merging, and chimera removal. A total of 2.1 million reads were assigned as prey sequences (Table S2) and we detected prey DNA in 61.7% of all buccal swab samples with an average of 16,855 prey reads per sample (Range: 527–177,653).

In Manx shearwater buccal swab samples (*n* = 169), fish prey was detected in 68.0% of cases using the FISH2_16S primer, including 66.7% of adult samples (*n* = 36) and 68.4% of chick samples (*n* = 133). In the fulmar samples (*n* = 32), fish prey was found in 28.1% of cases overall, with detection in 27.3% of adult samples (*n* = 22) and 30% of chick samples (*n* = 10).

Across all samples of both species, we detected fourteen fish taxa (Table S3). We identified eleven prey items to species level and three to genus level: sandeels (*Ammodytes*), true mackerels (*Scomber*) and sticklebacks (*Gasterosteus*).

**FIGURE 2:**
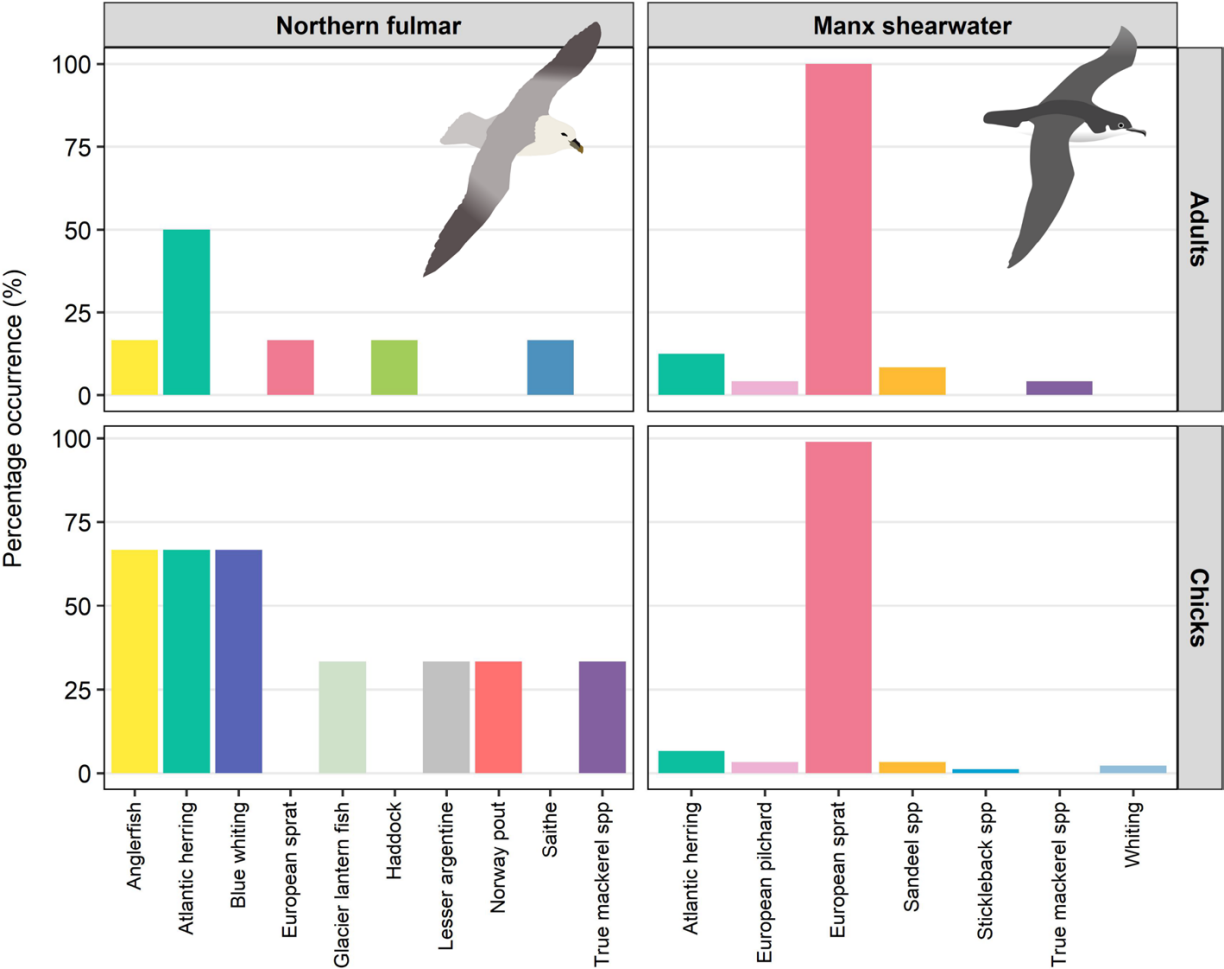
Percentage Occurrence (PO) of fish prey taxa in buccal swab samples; fulmar adult (*n* = 6), fulmar chick (*n* = 3), Manx shearwater adult (*n* = 24) and Manx shearwater chick (*n* = 91).

**FIGURE 3:**
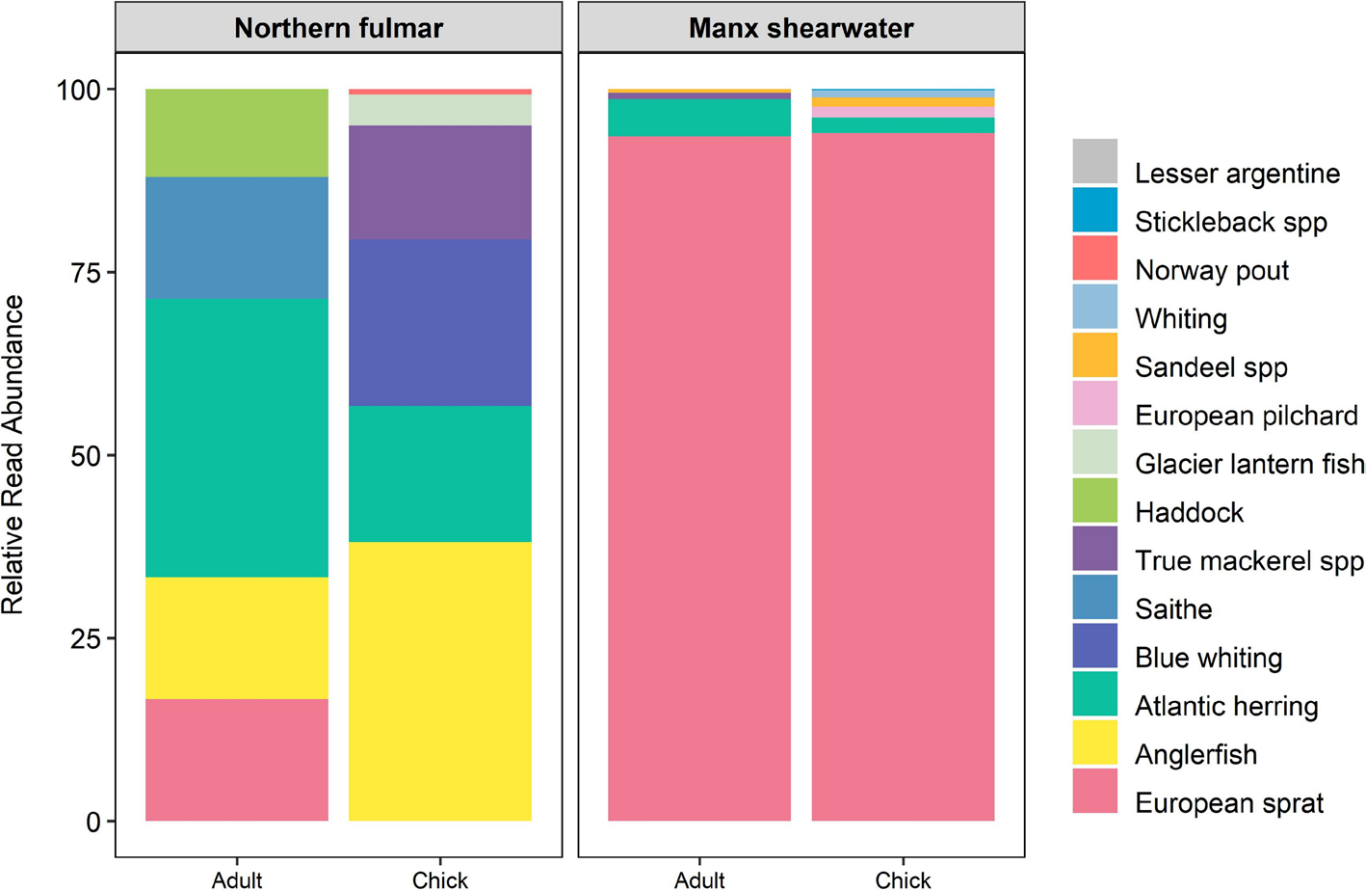
Relative read abundance (RRA) of fish prey taxa in buccal swab samples; fulmar adult (*n* = 6), fulmar chick (*n* = 3), Manx shearwater adult (*n* = 24), Manx shearwater chick (*n* = 91).

**FIGURE 4:**
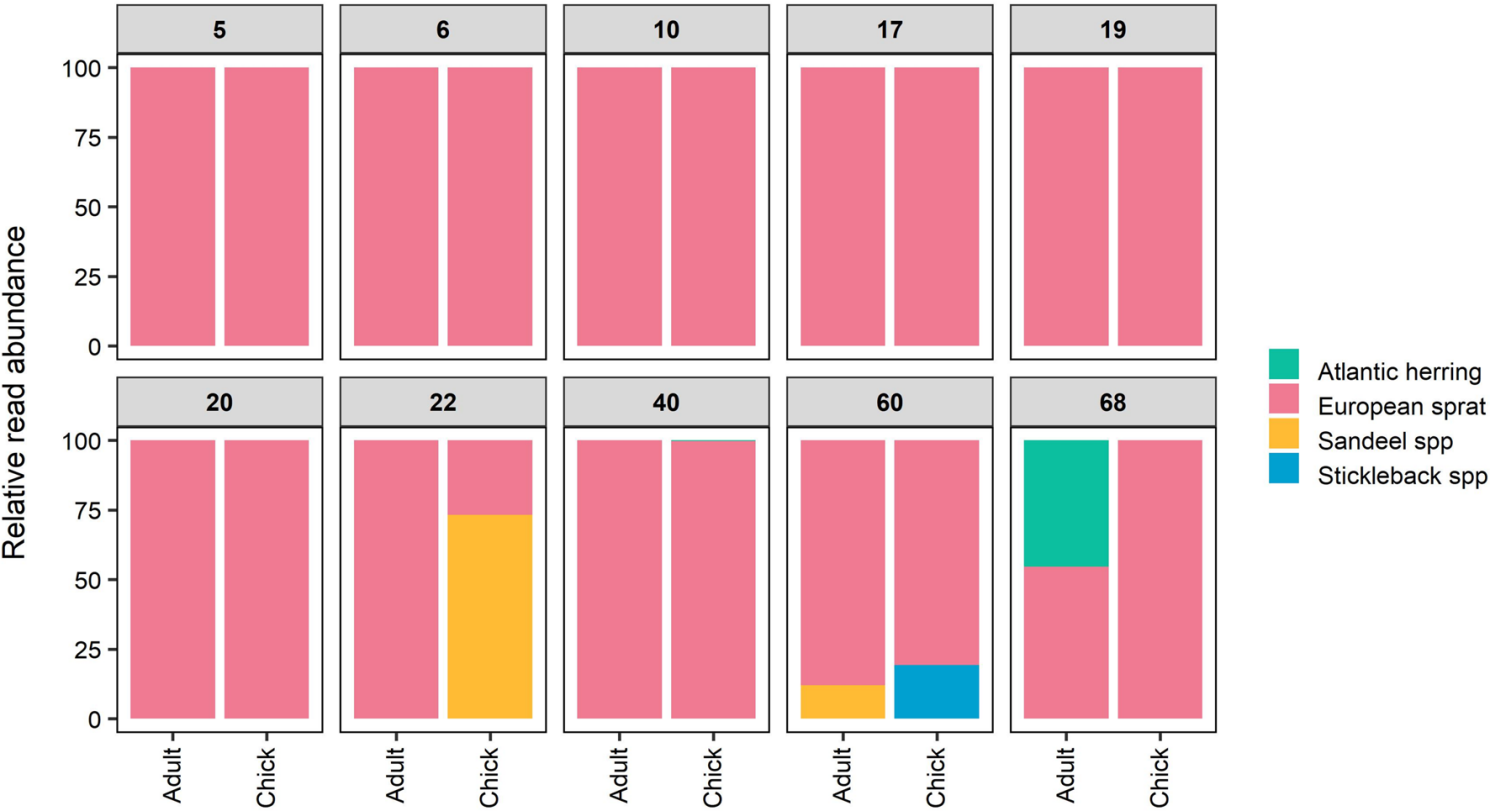
Relative read abundance (RRA) of prey taxa showing similarity between paired adult and chick Manx shearwater samples (*n* = 10). Panel headers represent burrow numbers.

Manx shearwater samples contained seven fish prey taxa (Figure 2). European sprat ( *Sprattus sprattus* ) made up 93.9% of all fish reads and was present in all samples except one. Atlantic herring ( *Clupea harengus* ) was the second most common Manx shearwater prey item, representing 2.7% fish reads and present in nine samples, followed by sandeels with 1.14% of fish reads in five samples (Figure 3). In adult samples, we detected five prey taxa with a mode of 1 prey taxon (Range: 1–3) per sample. Across all Manx shearwater chick samples, we detected six prey taxa with a mode of 1 prey taxon (Range: 1–2) per sample (Figure S1). Paired samples collected from Manx shearwater adults and their chicks on the same night showed notable similarity, largely driven by seven pairs in which only European sprat was detected. In contrast, in other paired samples where additional prey taxa were detected, a consistent pattern of shared prey between adults and chicks was less clear (Figure 4).

Fulmar samples contained ten different prey items (Figure 2) with Atlantic herring the most common prey item representing 31.5% of total reads and present in five samples. The second most common prey item was anglerfish ( *Lophius piscatorius* ) with 23.8% prey species reads in three samples, followed by blue whiting ( *Micromesistius poutassou* ) represented by 7.6% of reads and present in two samples (Figure 3). In adult samples, we detected five prey taxa with a mode of 1 prey taxon (Range: 1–2) per sample. In fulmar chick samples, we detected seven prey taxa with a mode of two prey taxa (Range: 2–5) per sample (Figure S2).

### 3.1 Cephalopod & Decapod

Whilst the cephalopod positive control was successfully amplified using the Ceph18S primer set, we detected no cephalopod prey DNA in any samples. Using the MiDeca primer set, we only detected decapod prey DNA in one Manx shearwater sample equating to 2.9% of total reads (Figure S3). Decapod prey comprised seven different species: hermit crab (*Anapagurus laevis*), ghost shrimp ( *Callianassa subterranea* ), brown shrimp ( *Crangon crangon* ), blue striped squat lobster ( *Galathea strigosa* ), wrinkled swimming crab ( *Liocarcinus corrugatus* ), couch rubble crab (*Monodaeus couchii*), long clawed porcelain crab ( *Pisidia longicornis* ) and one family of mud shrimp (*Upogebiidae* spp.) which accounted for 88.5% of all decapod reads (Table S4).

**FIGURE 5:**
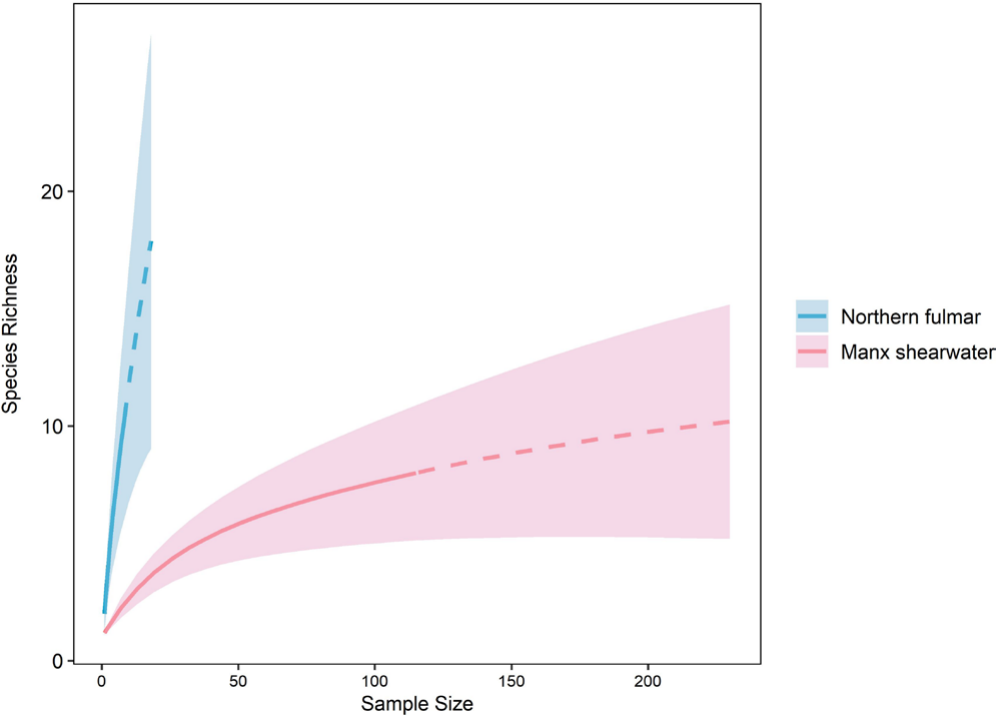
Species accumulation curve showing observed (solid line) and extrapolated (dashed line) cumulative species richness as a function of number of samples for fulmars (*n* = 9, extrapolated to 18 samples) and Manx shearwaters (*n* = 115, extrapolated to 230 samples). Error bars indicate standard error after 100 random permutations.

Species accumulation curves suggest that 115 samples was sufficient to describe the diet of Manx shearwaters as the curve is close to plateauing. On the other hand, nine samples were insufficient to capture the broad dietary diversity of fulmars (Figure 5).

## 4 Discussion

This study represents the first application of buccal swabbing for DNA‐based dietary analysis in seabirds, offering a minimally invasive method to investigate diet in these wide‐ranging species. By applying this technique to two Procellariiform species, we found low dietary diversity in Manx shearwaters and identified fishery discard species in the diet of fulmar adults and chicks. Sampling during incubation and chick‐provisioning periods, as well as from chicks, demonstrated this technique is suitable for dietary studies across different breeding stages and has potential to also be used in the non‐breeding season.

### 4.1 Manx Shearwaters

The overall dietary diversity of Manx shearwaters was limited, with seven fish taxa identified in 115 samples, while an additional eight decapod taxa were found in a single sample. Species accumulation curves suggested sample size was sufficient to fully capture dietary diversity. European sprat was identified as the most important prey taxa for Manx shearwaters, followed by Atlantic herring and sandeels. The most detected prey taxa in this study are consistent with those detected by DNA metabarcoding of faecal samples in the diet of pre‐laying and incubating birds on Skomer (Siddiqi‐Davies et al. 2025) and stomach content analysis of chick‐provisioning birds on Rhum (Thompson 1987). This reinforces the importance of these prey taxa for Manx shearwaters throughout the breeding season. We also detected four additional taxa not previously recorded in the diet of Manx shearwaters: European pilchard ( *Sardina pilchardus* ) was detected in four samples, whiting ( *Merlangius merlangus* ) in two samples whilst true mackerels (*Scomber*) and stickleback (*Gasterosteus*) were detected in one adult and one chick sample respectively.

The dominance of European sprat in Manx shearwater diet was notable and raises important questions about the foraging behaviour of this seabird species. One interpretation is that shearwaters exhibit dietary specialism on sprat, at least during the chick rearing period. On the one hand, this result is surprising given the extremely large spatial range over which breeding Manx shearwaters are known to forage (Harris et al. 2025). However, despite seasonal fluctuations in energy content, sprat have significantly higher calorific values and protein content when compared to herring, sandeels, and other forage fish species of a similar size (Harris and Hislop 1978; Hislop et al. 1991; Wanless et al. 2005). This suggests Manx shearwaters may preferentially target the most energetically profitable prey type. Alternatively, the high prevalence of sprat may indicate that shearwaters have an opportunistic and flexible foraging strategy, selecting prey based on local abundance. The sampling period may have coincided with seasonal life cycle events of sprat, making them disproportionality available to shearwaters and leading to their frequent detection in the diet. We note that our diet samples were collected from over 1 month only during 2024, and at one colony, and we strongly recommend future studies increase sample size over both time and space to fully explore this apparent reliance on sprat in Manx shearwater diet.

While adult seabirds are highly mobile and often difficult to capture, their nestlings are typically more readily accessible. Nestling seabirds are reliant on their parents for prey, such that collecting samples from seabird chicks may enable representative information on adult seabird diet to be gathered more easily (Carey 2009; Kennerley et al. 2024). At the population level, the prey species detected in adult Manx shearwaters were highly similar to those detected in chick samples. This indicates that chick samples reliably represent population‐level diets of both adults and chicks during this period. When comparing samples from individual pairs of Manx shearwater adults and chicks, there was high similarity within adult and chick pairs due to the uniformity of sprat. There were only three pairs where other prey taxa were detected, which were not consistent within adult and chick paired samples. Understanding the persistence of prey DNA in the buccal cavity of seabirds is crucial to interpreting this result, however we are unaware of any literature on this topic. Given that dietary DNA is known to persist in seabird faeces for up to 4 days (Deagle et al. 2010), it is plausible that both adult and chick swabs have also detected residual prey DNA from previous meals. Further work is required to determine whether chick samples accurately represent the contents of the last meal provided by a parent and if they can serve as a reliable proxy for chick‐provisioning adults at the individual level.

### 4.2 Northern Fulmars

Ten different prey taxa were identified across all fulmar samples. However, prey species accumulation curves (Figure 4) indicate that our small sample size was insufficient to fully capture fulmar dietary diversity. Atlantic herring and anglerfish were the most important prey taxa identified for both adults and chicks representing the highest numbers of relative reads and occurring in the greatest number of samples. Notably, anglerfish, one of the most important prey types, is previously undocumented in the diet of fulmars, as is lesser argentine ( *Argentina sphyraena* ) (Furness and Todd 1984; Phillips et al. 1999; Ojowski et al. 2001; Garthe et al. 2004; Mallory et al. 2010). Two additional prey species, five‐bearded rockling ( *Ciliata Mustela* ) and common ling ( *Molva molva* ), were detected in samples which did not meet a sufficient sequencing depth of 500 reads per sample. Although these represent new prey species for fulmars, the samples were excluded from further analyses due to insufficient read depth.

Fulmars are dietary opportunists and are known to associate with fishing vessels (Phillips et al. 1999; Darby et al. 2021). Many of the prey taxa identified are either demersal species, living at depths greater than fulmars' diving abilities, or are commercially valuable, suggesting these species have been scavenged as fishery discards. Prey items likely obtained from fisheries discards include the Gadids (haddock *Melanogrammus aeglefinus* , blue whiting, saithe *Pollachius virens* , Norway pout *Trisopterus esmarkii* ); the family Argentinidae (lesser argentine); the true mackerels and anglerfish (Camphuysen and van Franeker 1997; Phillips et al. 1999; Danielsen et al. 2010). On the other hand, the clupeids (European sprat & Atlantic herring) likely represent natural prey (Ojowski et al. 2001). Glacier lantern fish ( *Benthosema glaciale* ) hold no significant commercial value but are known to inhabit depths of 150–500 m; their diel vertical migration may increase their vulnerability to predation by surface‐feeding fulmars (Danielsen et al. 2010). Our results highlight the potential of this technique to enhance our understanding of the reliance of fulmars on fishery discards. By integrating individual‐level diet sampling and biologging, this approach could reveal whether scavenging behaviour is associated with specific vessel types, geographic location, or whether there is intraspecific variation in discard use.

### 4.3 Assessment of the Method

We detected no cephalopod DNA in any samples. Previous studies, using stomach content analysis or stable isotope analysis, have concluded squid, in particular the family Ommastrephidae, constitutes a major prey type for both Manx shearwaters and fulmars. However, evidence suggests that both species transition to feeding on higher trophic levels, with greater calorific values, during the breeding season (Thompson 1987; Furness 1994; Phillips et al. 1999; Ojowski et al. 2001; Mallory et al. 2010). There is no evidence to suggest that cephalopod DNA degrades faster than fish DNA (Deagle and Tollit 2007; McInnes, Alderman, et al. 2017) or has gone undetected in this study; however, we acknowledge that multiplexing of primer sets may reduce amplification efficiency. Accordingly, this research suggests both species have a primarily piscivorous diet during our study seasons. Decapod DNA was only present in one Manx shearwater sample; however, this sample exhibited notable diversity with eight distinct taxa detected. On the one hand, the presence of decapod DNA in this single sample may be the result of secondary predation, due to higher‐than‐average sequencing depth (Thompson 1987; Bowser et al. 2013; Siddiqi‐Davies et al. 2025). However, if this was the case, we might reasonably expect decapod DNA to be consistently present in more samples containing fish DNA. Another potential explanation is that the presence of decapod DNA represents true predation, and Manx shearwaters occasionally supplement their primarily fish‐based diet with other prey types, such as decapods. We did not detect decapod DNA in any fulmar samples, which is somewhat surprising given that voluntary regurgitations of fulmars during handling sometimes contain fragments of decapod hard parts; although the absence of decapod DNA may be attributable to the small number of fulmar samples. Additionally, we recognize that detection probabilities likely differ among fish, cephalopods, and decapods, as the exoskeleton of many decapod taxa has been shown to reduce DNA shedding into the environment (Tréguier et al. 2014; Forsström and Vasemägi 2016). This may have hindered the detection of decapod DNA in our samples compared to that of soft‐bodied organisms.

As with many dietary DNA sampling techniques, buccal swabbing results in large amounts of host DNA, and so careful primer design is essential to avoid overwhelming amplification of host DNA (Paprocki et al. 2024). In this study, host DNA accounted for an average of 89.2% reads per sample. Although blocking primers are commonly used to overcome the issue of host amplification (Nota et al. 2019), they can inadvertently bias prey detection and were not available for either of our study species (McInnes, Jarman, et al. 2017; Kleinschmidt et al. 2019). As developing blocking primers was beyond the scope of this study, we instead aimed to reduce the impact of host amplification by increasing sequencing depth. However, we recognize the use of blocking primers may be a useful strategy to reduce host DNA in future studies.

Across both species, 61.7% of samples contained prey DNA. Despite the relatively low success rate of fulmar samples, the proportion of buccal swab samples yielding dietary DNA in this study was greater than or equal to, that of faecal samples in several comparable Procellariiform diet studies (McInnes, Alderman, et al. 2017; McInnes, Jarman, et al. 2017; Campioni et al. 2023; Siddiqi‐Davies et al. 2025) while retaining the advantage of allowing targeted individual sampling. A greater proportion of Manx shearwater samples yielded prey DNA compared to fulmar samples. This difference may be due to differences in enzymatic or microbial activity between the species which act to degrade DNA within the mouth (Monge et al. 2020). However, adult fulmars were sampled opportunistically and the interval between feeding and swabbing may have been up to 10 days due to prolonged incubation stints (Mallory et al. 2008). Fulmar chicks were swabbed opportunistically during late chick‐rearing. Even when chicks are small, they may experience a maximum of 16‐h between meals (Hamer and Thompson 1997) but, by the sampling period in mid‐August, they are likely fed even less frequently. In contrast, Manx shearwaters were swabbed no more than 1 h after an adult had regurgitated a meal for its chick. We therefore suggest that differences in amplification success are more likely due to the time interval between regurgitation and sample collection rather than species‐specific differences in DNA degradation. The results of this study support the conclusions of Komura et al. (2018) and Siddiqi‐Davies et al. (2025) who suggest DNA degradation during long trips or incubation fasts reduces the amount of detectable prey DNA. We further emphasise the importance of minimising the time elapsed since feeding when designing sampling protocols for this technique and would recommend future studies investigate the persistence of detectable dietary DNA in the buccal cavity of birds.

## 5 Conclusion

The application of buccal swabbing to DNA‐based dietary analysis offers significant advancements for seabird diet studies in situations where visual observation is not possible, including both incubating birds and regurgitating individuals during chick provisioning, provided birds can be sampled shortly after feeding or regurgitation. We have demonstrated that, for chick‐provisioning Manx shearwaters, chick samples, which are often easier to obtain, can reliably represent a species' diet at the population level. This may prove especially useful for species of conservation concern or those where adults are particularly sensitive to disturbance.

From a conservation perspective, our findings indicate the potential for these techniques to contribute to a better understanding of the drivers of population change in both our study species and other Procellariiformes. This is particularly relevant for fulmars given recently reported population declines in UK waters (Dunn et al. 2023) and uncertainty over the extent to which declines may be driven by interactions with fisheries (Darby et al. 2021; Clegg et al. 2024) and/or responses to natural variation in food supplies (Thompson and Ollason 2001; Darby et al. 2023). For Manx shearwaters, these preliminary results indicate dietary specialisation, with a potential reliance on a single prey species during the chick‐provisioning period, although we acknowledge multi‐year data are needed to account for interannual variability in prey availability and foraging behaviour. When combined with bio‐logging technologies, buccal swabbing for DNA metabarcoding could help identify critical feeding areas and prey stocks for protected seabirds and improve our understanding of how fisheries management could support conservation efforts. The application of this technique could extend to other marine top predators and highly mobile or wide‐ranging species, offering a valuable tool to inform marine conservation.

We have demonstrated that buccal swabbing and DNA metabarcoding is an informative technique for assessing diet in seabird species, offering a viable alternative to faecal samples and allowing researchers to target specific individuals for diet sampling. These findings underscore the potential of buccal swabbing for DNA‐based dietary analysis to enhance our understanding of seabird foraging ecology and inform conservation strategies.

## Author Contributions

**Lauren G. Evans:** conceptualization (equal), data curation (lead), formal analysis (lead), investigation (lead), methodology (equal), project administration (lead), resources (equal), visualization (lead), writing – original draft (lead), writing – review and editing (equal). **Stephanie M. Harris:** conceptualization (equal), funding acquisition (supporting), investigation (supporting), methodology (supporting), supervision (supporting), writing – review and editing (equal). **Paul M. Thompson:** investigation (supporting), project administration (supporting), resources (supporting), writing – review and editing (equal). **Amy Ellison:** conceptualization (equal), formal analysis (supporting), funding acquisition (supporting), investigation (supporting), methodology (equal), project administration (supporting), resources (equal), supervision (equal), writing – review and editing (equal). **Line S. Cordes:** conceptualization (equal), funding acquisition (lead), methodology (equal), project administration (supporting), supervision (equal), visualization (equal), writing – review and editing (equal).

## Conflicts of Interest

The authors declare no conflicts of interest.

## Supporting information


Data S1.

## Data Availability

Raw sequence data is available at European Nucleotide Archive (project accession number PRJEB88873). Metadata and all R scripts are available as Supporting Information.
