# Supplementary material for: Advancing Seabird Diet Studies Through Buccal Swabbing for DNA Metabarcoding
Source: Ecol Evol. 2025 Jul 9;15(7):e71606. doi: 10.1002/ece3.71606 (PMC12238773; doi:10.1002/ece3.71606)
Supplement: Supplementary file 1 — Data S1. [file ECE3-15-e71606-s001.zip › ece371606-sup-0008-TablesS1-S4.docx]

**Appendix 1:**

Table 1: Primer sequences used to amplify Northern fulmar and Manx shearwater buccal samples for next-generation sequencing.

| **Reference** | (Deagle et al., 2009; Kleinschmidt et al., 2019; Waap, 2015) | (De Jonge et al., 2021) | (Komai et al., 2019) |
| --- | --- | --- | --- |
| **Annealing temperature (°C)** | 58 | 62 | 60 |
| **Approx. produce size incl. primer sequence (bp)** | 264 | 200 | 164 |
| **Rev Sequence** | GCTGTTATCCCTRGRGTAA | GCACTTAACCGACCGTCGAC | ACGCTGTTATCCCTAAAG |
| **Fwd Sequence** | CGAGAAGACCCTDTGRAG | CGCGGCGCTACATATTAGAC | GGACGATAAGACCCTATAAA |
| **Primer name** | Fish2_16S | Ceph18S | MiDeca |
| **Gene** | 16S rRNA | 18S rRNA | 16S rRNA |
| **Target** | Fish | Cephalopod | Decapod |

Table 2: Number of reads passing each stage of the DADA2 pipeline; filtering, denoising, merging and chimera removal for Fish2, Ceph18S and MiDeca primer sets.

| **Prey seq** | 2090059 | 0 | 28420 | 2118479 |
| --- | --- | --- | --- | --- |
| **No Chimera** | 27854307 | 259230 | 994684 | 29108221 |
| **Merged** | 44539883 | 310490 | 1027709 | 45878082 |
| **Denoised Rev** | 47068370 | 315306 | 1034295 | 48417971 |
| **Denoised Fwd** | 47070968 | 316973 | 1034312 | 48422253 |
| **Filtered** | 47109789 | 293581 | 994404 | 48397774 |
| **Raw** | 63783856 | 322934 | 1169313 | 65276103 |
|  | **Fish2** | **Ceph18S** | **MiDeca** | **Total** |

Table 3: List of fish prey taxa identified across all Northern fulmar and Manx shearwater samples, including family, scientific name, and common name of each taxon.

| **Family** | **Scientific Name** | **Common Name** |
| --- | --- | --- |
| Ammodytidae | *Ammodytes* | sandeels |
| Argentinidae | *Argentina sphyraena* | Lesser argentine |
| Myctophidae | *Benthosema glaciale* | Glacier lantern fish |
| Clupeidae | *Clupea harengus* | Atlantic herring |
| Gasterosteidae | *Gastersteus* | sticklebacks |
| Lophidae | *Lophius piscatorius* | Anglerfish |
| Gadidae | *Melanogrammus aeglefinus* | Haddock |
| Gadidae | *Merlangius merlangus* | Whiting |
| Gadidae | *Micromesistius poutassou* | Blue whiting |
| Gadidae | *Pollachius virens* | Saithe |
| Clupeidae | *Sardina pilchardus* | European pilchard |
| Scombidae | *Scomber* | true mackerels |
| Clupeidae | *Sprattus sprattus* | European sprat |
| Gadidae | *Trisopterus esmarkii* | Norway pout |

Table 4: List of decapod taxa identified in one adult Manx shearwater sample including family, scientific name, and common name of each taxon.

| **Family** | **Scientific Name** | **Common Name** |
| --- | --- | --- |
| Paguridae | *Anapagurus laevis* | Hermit crab |
| Callianassidae | *Callianassa subterranea* | Ghost shrimp |
| Crangonidae | *Crangon crangon* | Brown shrimp |
| Galatheidae | *Galathea strigosa* | Blue striped squat lobster |
| Polybiidae | *Liocarcinus corrugatus* | Wrinkled swimming crab |
| Xanthidae | *Monodaeus couchii* | Couch rubble crab |
| Porcellanidae | *Pisidia longicornis* | Long clawed porcelain crab |
| Upogebiidae | Upogebiidae | Mud shrimp spp |
