# Supplementary figures and images for: Advancing Seabird Diet Studies Through Buccal Swabbing for DNA Metabarcoding
Source: Ecol Evol. 2025 Jul 9;15(7):e71606. doi: 10.1002/ece3.71606 (PMC12238773; doi:10.1002/ece3.71606)

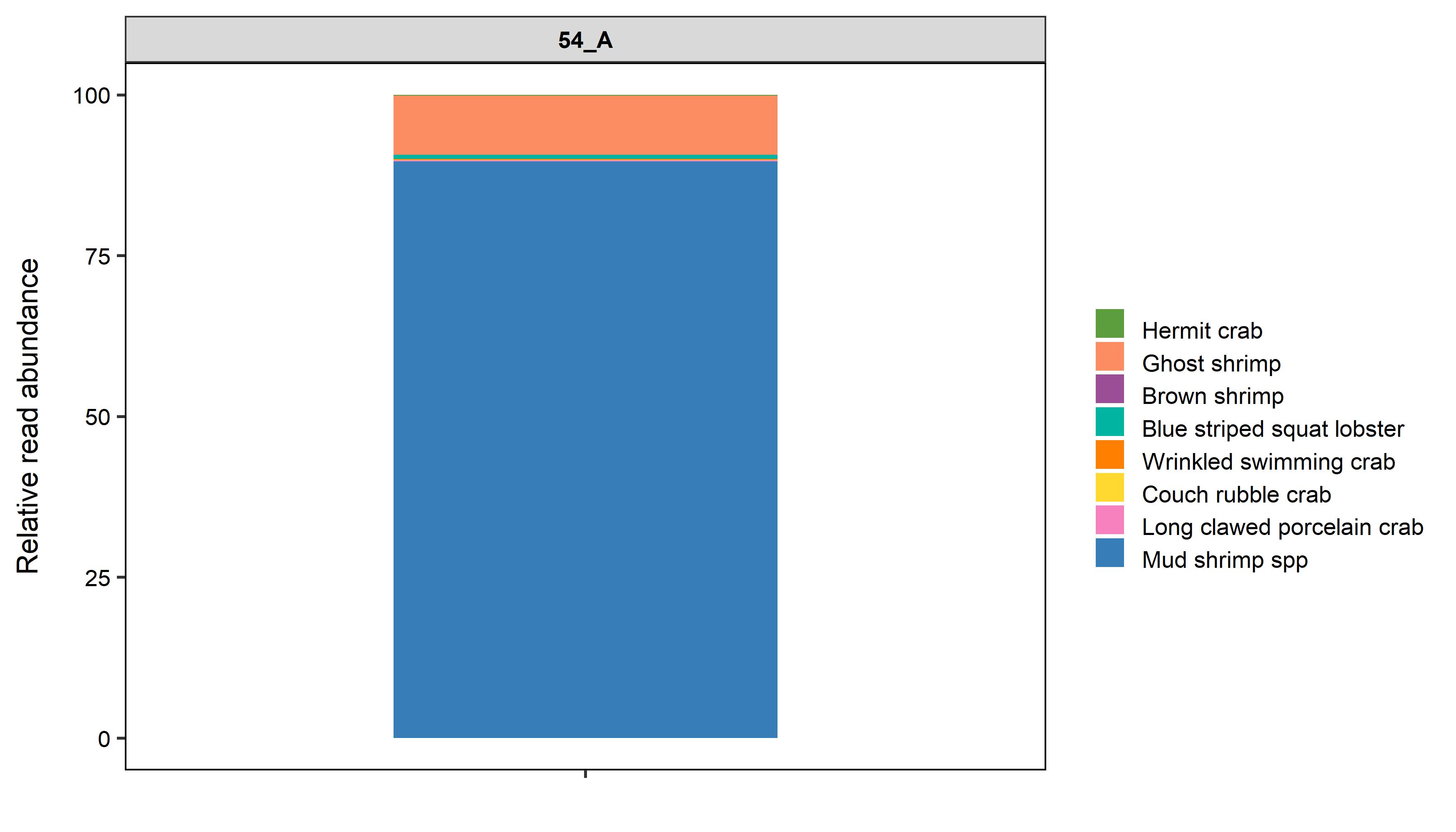

Supplement: Supplementary file 1 — Data S1. [file ECE3-15-e71606-s001.zip › ece371606-sup-0003-FigureS3.tiff]

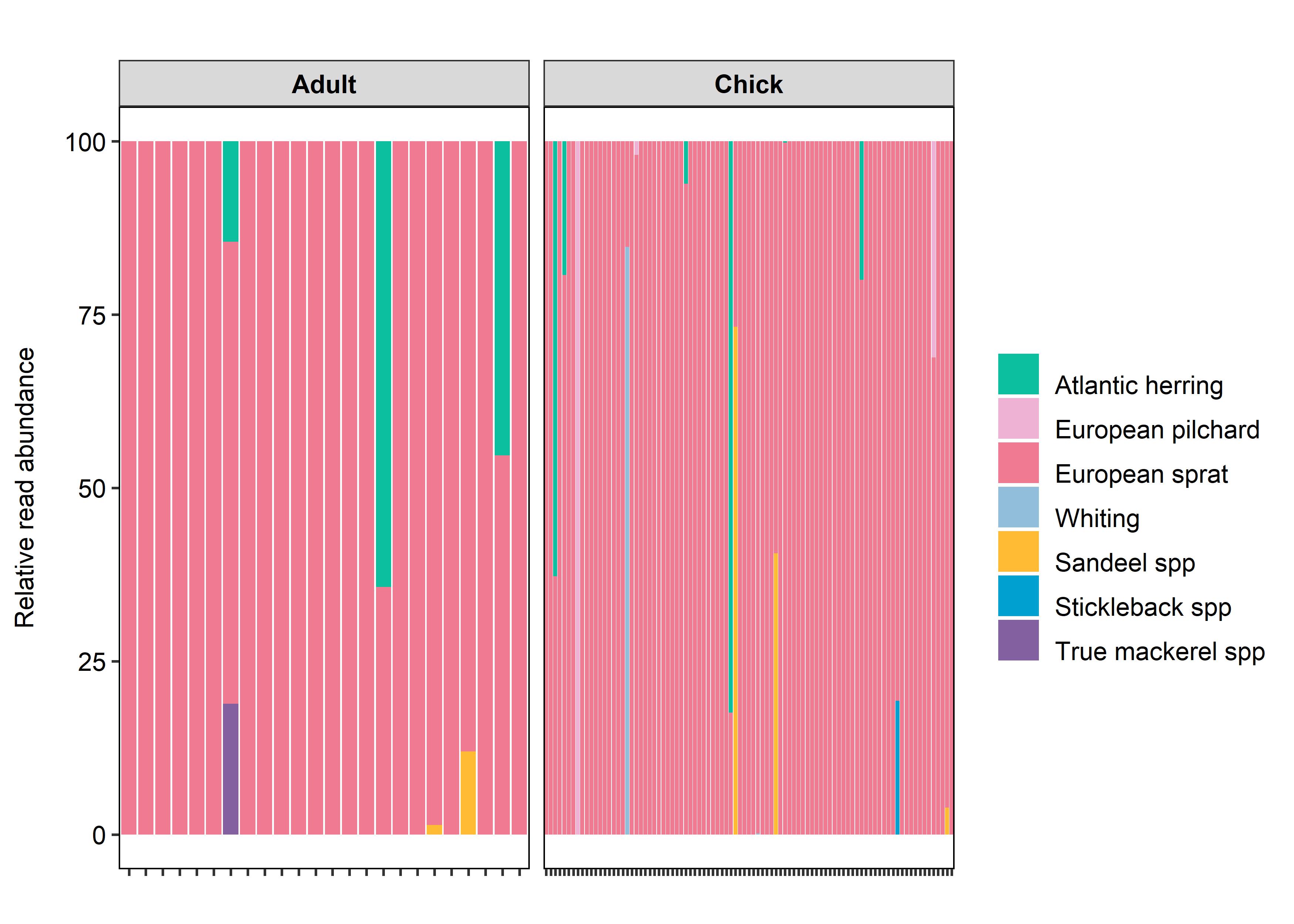

Supplement: Supplementary file 1 — Data S1. [file ECE3-15-e71606-s001.zip › ece371606-sup-0001-FigureS1.tiff]

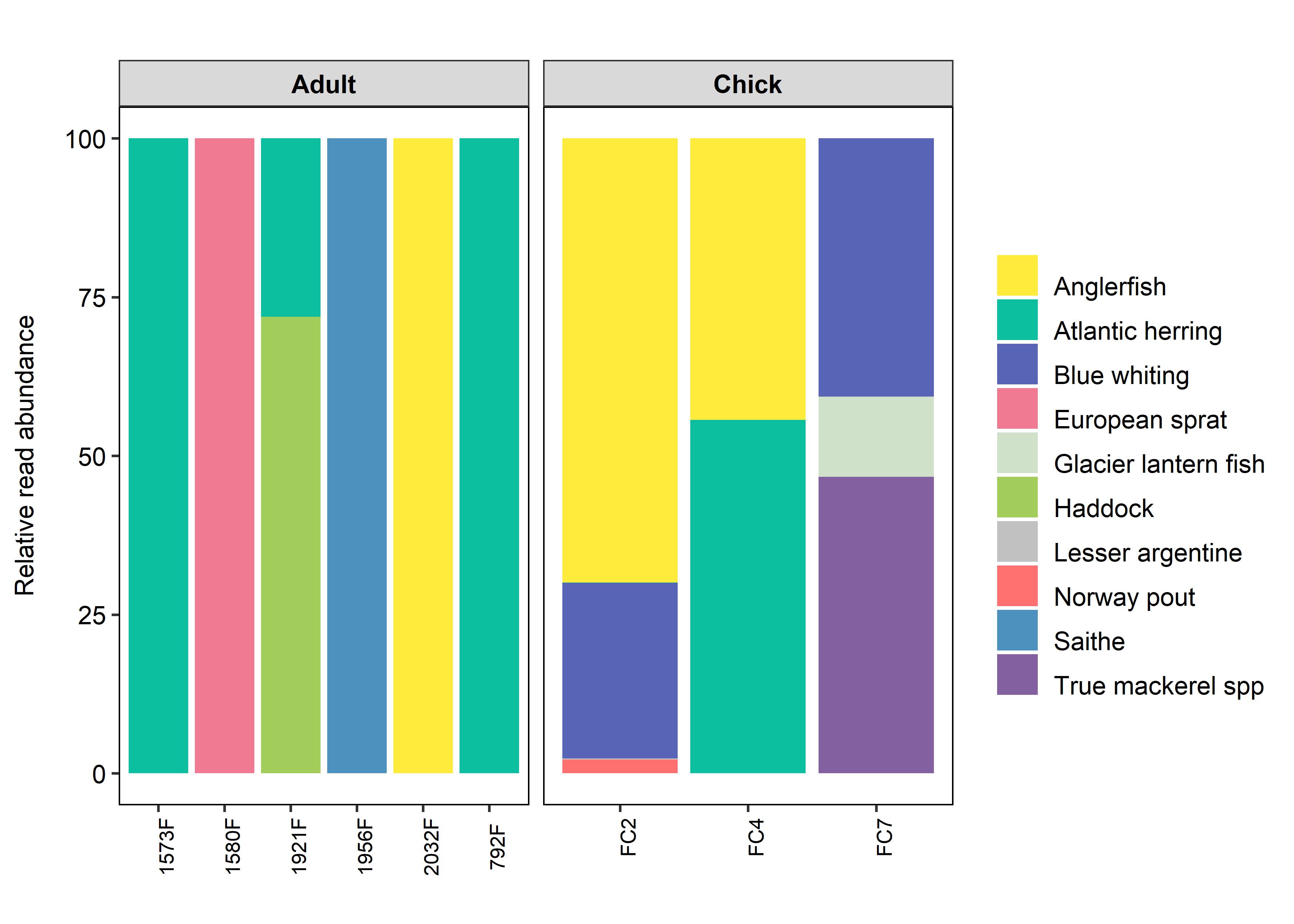

Supplement: Supplementary file 1 — Data S1. [file ECE3-15-e71606-s001.zip › ece371606-sup-0002-FigureS2.tiff]
